# In Vitro Evaluation of Biomaterials for Vocal Fold Injection: A Systematic Review

**DOI:** 10.3390/polym13162619

**Published:** 2021-08-06

**Authors:** Ng Wan-Chiew, Marina Mat Baki, Mh Busra Fauzi, Yogeswaran Lokanathan, Mawaddah Azman

**Affiliations:** 1 Department of Otorhinolaryngology—Head and Neck Surgery, Faculty of Medicine, Universiti Kebangsaan Malaysia, Kuala Lumpur 56000, Malaysia; wanchiew1997@gmail.com (N.W.-C.); marinamatbaki@ppukm.ukm.edu.my (M.M.B.); 2 Centre for Tissue Engineering and Regenerative Medicine, Faculty of Medicine, Universiti Kebangsaan Malaysia, Kuala Lumpur 56000, Malaysia; fauzibusra@ukm.edu.my (M.B.F.); lyoges@ppukm.ukm.edu.my (Y.L.)

**Keywords:** vocal fold augmentation, functional voice disorder, preliminary study, characterization, material

## Abstract

Vocal fold injection is a preferred treatment in glottic insufficiency because it is relatively quick and cost-saving. However, researchers have yet to discover the ideal biomaterial with properties suitable for human vocal fold application. The current systematic review employing PRISMA guidelines summarizes and discusses the available evidence related to outcome measures used to characterize novel biomaterials in the development phase. The literature search of related articles published within January 2010 to March 2021 was conducted using Scopus, Web of Science (WoS), Google Scholar and PubMed databases. The search identified 6240 potentially relevant records, which were screened and appraised to include 15 relevant articles based on the inclusion and exclusion criteria. The current study highlights that the characterization methods were inconsistent throughout the different studies. While rheologic outcome measures (viscosity, elasticity and shear) were most widely utilized, there appear to be no target or reference values. Outcome measures such as cellular response and biodegradation should be prioritized as they could mitigate the clinical drawbacks of currently available biomaterials. The review suggests future studies to prioritize characterization of the viscoelasticity (to improve voice outcomes), inflammatory response (to reduce side effects) and biodegradation (to improve longevity) profiles of newly developed biomaterials.

## 1. Introduction

Population-based studies among subjects over 60 years old or more reported up to 29% prevalence of vocal disorders, mainly from glottic insufficiency [1]. Incomplete closure of the vocal fold, namely glottic insufficiency, results in a breathy voice and risk of aspiration [2]. These symptoms are commonly caused by unilateral vocal fold paralysis or paresis and are mainly subsequent to surgery. The recurrent laryngeal nerve that supplies most intrinsic laryngeal muscles is at risk in surgeries of the thyroid gland, neck, trachea and esophagus [3]. Other risk factors include poor regeneration due to senescence, smoking and systemic disease [4]. Young recurrent laryngeal nerve (RLN) tissue appeared more elastic to strain, while adolescent RLN tissue was stiffer and tended to break with strain [5]. In treating glottic insufficiency, vocal fold injection is preferred as it is more cost-effective and time-saving [6,7]. Currently available biomaterials for vocal fold injection include bovine and porcine gelatin, carboxymethylcellulose, calcium hydroxylapatite (CaHA), autologous fat, collagen-based and hyaluronic acid (HA)-based hydrogels [8]. Vocal fold injection is also commonly performed concomitantly with nonselective laryngeal reinnervation [9].

In developing a suitable biomaterial for vocal fold augmentation, the biochemical properties of the vocal fold need to be respected. This is because the intervention has the potential to interrupt the microenvironment of the vocal fold, which eventually affects the quality of voice production [10]. Vocal folds are two bands of complex tissue that serve multiple roles, including respiration, voice production and airway protection [11]. The complex tissue consists of superficial and deep layers separated by the lamina propria [12]. Additionally, the lamina propria is further divided into three parts: (1) superficial, (2) intermediate and (3) deep layers [13]. The myoelastic-aerodynamic theory explains the unique characteristics of the vocal fold and the neuromuscular control that permits phonation [14]. The superomedial aspect of the superficial layer of the vocal fold is softer compared to its inferomedial surface [15]. The viscoelasticity of the vocal fold is primarily determined by the composition of the extracellular matrix (ECM) [16]. The ECM in the vocal fold consists of collagen and elastin as the fibrous proteins proteoglycan and glycoprotein, as interstitial proteins. These proteins play vital roles in maintaining the viscoelasticity of the human vocal fold [17]. Elastin enables vocal fold tissue to possess elastic properties and is more abundant in the superficial parts of the lamina propria than its deeper layers [18]. On the other hand, collagen in the vocal fold functions as a mechanical support to withstand high-frequency vibration and stretching [13]. The abundance of collagen was found in the deep layer of the lamina propria. Due to its non-linear properties, it remains unclear whether current quantification methods used by recent studies suit the viscoelasticity of native vocal fold tissue.

The vocal fold is very sensitive towards any intrusion. A previous study has explained that airborne particulate matter significantly upregulated interleukin-6 (IL-6) and IL-1β which mounts a pro-inflammatory response. In addition, these particles could initiate mitogen-activated protein kinase (MAPK) and necrosis factor kappa beta (NF-ĸB) pathways [19]. Furthermore, a recent clinical study reported that the most frequent complication after vocal fold injection was inflammation at the injection site [20]. Activation of inflammation in the vocal fold increases fibrosis formation leading to vocal fold scarring, subsequently causing dysphonia [21,22]. Therefore, unregulated inflammatory response is shown to be an adverse event in the vocal fold. The molecules involved in the inflammatory response in the vocal fold have been extensively studied for at least a decade [23,24]. The consistency of studies in quantifying IL-1β, NF-ĸB, tumor necrosis factor-alpha (TNF-α), interferon-gamma (IFN-γ), transforming growth factor-beta (TGF-β), cyclooxygenase (COX2), HA and procollagen expression indicated that these molecules are closely related to inflammation in the vocal fold [25]. Inflammation starts with the accumulation of IL-1β, NF-ĸB and TNF-α, activating fibroblasts and promoting collagen deposition, leading to scar formation [24].

Multiple biomaterials have been used in vocal fold injections but only CaHA and autologous fat have a long duration without resorption [26]. However, application of CaHA imposed the risk of migration and granuloma formation [27]. Even though HA possesses the most favorable properties, its inconsistent resorption is the main drawback [28,29]. Well-developed biomaterials should be tolerated by the host immune system and non-antigenic surface receptors even after degradation [30,31]. More prolonged effects of injection and biocompatibility are the main desired outcomes in the development of future injectable biomaterials [32,33]. Before further investigation up to in vivo and clinical study, the optimization stage of biomaterials is performed. However, the parameters to be assessed during preliminary study are not clear. Therefore, the myriad applications of biomaterials in glottic insufficiency research have been chosen for discussion in this review. A literature search was performed through electronic databases to identify characterization of biomaterials and in vitro studies performed on the application of biomaterials in acute and chronic glottic insufficiency. The objective of this review is to describe the outcome measures used to study the various physicomechanical characteristics of biomaterials developed for the vocal fold. Additionally, the review will provide insight on which aspects should be focused on in future studies.

## 2. Materials and Methods

### 2.1. Search Strategy

A systematic review of the literature was performed to determine relevant studies reporting the characterization of biomaterials and in vitro studies to test their applicability as vocal fold injections for the glottic insufficiency condition. The systematic review was structured based on PRISMA guidelines to ensure the quality and transparency of this review [34]. A total of four databases includes Scopus (Elsevier, Amsterdam, NH, The Netherlands), ISI Web of Science (WoS) (Clarivate Analytics, Philadephia, PA, USA), PubMed (National left for Biotechnology Information, NCBI, Bethesda, MD, USA) and Google Scholar (Mountain View, CA, USA) were used to search relevant articles within the last eleven years (from January 2010 to March 2021). The article searching process was guided by the focus question formulated using the PICO strategy whereby population (P) was laboratory study on biomaterials for vocal fold injection; intervention (I) was outcome measure used to characterize the biomaterials; comparison (C) with other biomaterials was not applicable; and outcome (O) was physicomechanical or cellular characteristics of the biomaterials studied. The searching process was performed by two independent reviewers.

The searching method was performed by two sets of keyword combinations: (1) biomaterial* (to obtain biomaterial or biomaterials) or material* (to obtain material or materials) or regenerative therap*(to obtain regenerative therapy or regenerative therapies) or hyaluronic acid* (to obtain hyaluronic acid or hyaluronic acids) and (2) vocal fold injection* (to obtain vocal fold injection or vocal fold injections) or glottic insufficienc* (to obtain glottic insufficiency or glottic insufficiencies) or vocal fold medialization* (to obtain vocal fold medialization or vocal fold medializations) or vocal fold augmentation* (to obtain vocal fold augmentation or vocal fold augmentations). The search strategy was performed for all databases following summary in Table 1.

### 2.2. Inclusion Criteria

Due to limited resources for translation, only English articles were included. Original research articles discussing the effects of biomaterials used in injection laryngoplasty with the main priority of glottic insufficiency applications were chosen. The studies that involved different types of biomaterials were included in this review. Studies on the cells involved during healing in glottic insufficiency including fibroblasts, endothelial cells and macrophages were also included.

### 2.3. Exclusion Criteria

The exclusion criteria included all secondary literature and any original articles solely involving in vivo and clinical stage study, articles written or submitted in languages other than English and studies focused on developing biomaterials using biological elements such as cells, growth factors, genes and tissue components derived from animals.

### 2.4. Data Extraction and Management

Articles were screened in three phases to fulfill part of this systematic review. The first step included screening of titles to remove titles that did not match the inclusion criteria. The second step included abstract screening of the remaining papers to further remove inappropriate articles based on the inclusion criteria. The last step included removing any papers that did not meet the inclusion criteria after full-text reading by two independent reviewers. This review could not be published on PROSPERO because it included in vitro studies.

### 2.5. Quality Assessment

The review was conducted in a methodological approach using the critical appraisal instrument for systematic reviews [35]. The primary and secondary reviewers discussed each item in the appraisal instrument for each study included in this review. All studies included were considered acceptable to the aims of this review in terms of the specific study characteristics. A prior discussion between the independent reviewers was performed to determine what constitutes acceptable levels of information to allocate a positive appraisal compared with negative, or “unclear” responses. We utilized an appraisal instrument consisting of 11 questions where each question was answered with “yes”, “no” or “unclear”.

## 3. Result

### 3.1. Searching Result

**Figure 1:**
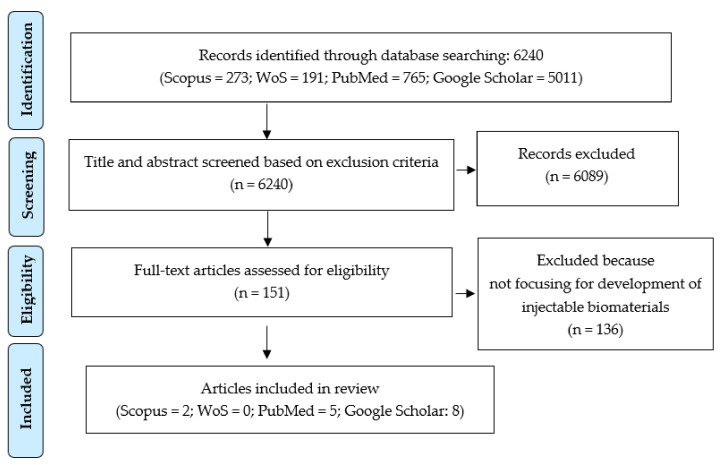
PRISMA flow diagram.

Total of 6240 articles were identified as potentially relevant. The first screening removed a total of 6089 articles which were non-original articles, not written or submitted in English, were duplicates or had a title or abstract that did not fit the inclusion criteria. From the remaining 151 articles, reviewers read the full text and 136 articles were removed as the articles did not fulfill the inclusion criteria. After selection, total of 15 articles were chosen for reviewing. A flow chart of the article selection process is shown in Figure 1.

### 3.2. Study Characteristics

Among the selected articles, all articles included parameter in characterizing physicomechanical properties of hydrogel. Total of 5 out of 15 studies involved cellular response study. Type of cells used for included in vitro study was listed in 3.5. Total of 7 out of 15 studies involved animal study but the data was not included as per exclusion criteria mentioned in 2.3. With that, the data were summarized into three different aspects including rheological properties, other characterization parameters such as pore size, particle size, injection force, swelling ratio, drug release test, morphology and gelation time and cellular response. All of the studies were published between January 2010 and March 2021. In general, ten studies aimed to develop new formulation of biomaterials [36,37,38,39,40,41,42,43,44,45]; three studies focused in improving or characterizing current biomaterials [46,47,48]; one study investigated the effect of the biomaterials toward inflammation [49]; three studies intended to improve fabrication methods in order to produce better biomaterials [36,46,50]. The biomaterials studied in the included articles were carbomer hydrogel, micronized dermal graft tissue, crosslinked HA, HA with gelatine hydrogel, CaHA, carboxymethylcellulose (CMC), bovine collagen, micronized alloderm (Cymetra) (Lifecell Corp, Branchburg, NJ, USA), HA gel, carboxylic and hydroxylic multi-walled functionalized carbon, unequal particle sized middle viscosity and low viscosity HA, Rofilan (Laborata es Filorga, Lisbonne, Paris, France),Radiesse (Merz, Franksville, WI, USA),Restylane (Galderma Laboratories, Fort Worth, TX, USA), dextran beads in HA microsphere (MP), polyethylene glycol-diamine (PEG) microparticles, gelatine methacrylate MP, HA methacrylate, semi-IPN MP, glycol chitosan hydrogel, pluronic F127 with collagen, HA with poly(ethylene glycol) diacrylate (PEGDA) crosslinkers, silk protein based in HA suspension, resilin-like-polypeptide hydrogel, PEG30 hydrogel and polydimethylsiloxane (PDMS) with polydopamine (PDA).

### 3.3. Rheological Properties

The most frequent characterization of biomaterials for vocal fold injection is rheological measurement; 14 out of 15 articles quantified this measurement with different rheometers and parameters. The outcome measures used include elastic shear modulus (G′), loss shear modulus (G″), loss tangent (ξ), dynamic viscosity (η′), Young modulus (E′), strain sweep and shear storage modulus. The proportional relationship between G′ and G″ with frequencies was investigated in most of the studies. Larger values of G′ compared to G″ show that the biomaterials possess more elastic behavior than viscous properties. Two studies tested viscoelasticity of the biomaterials at high frequencies, up to 2000 Hz and 4000 Hz, whereas in other studies the tested range was 0.1 Hz to 250 Hz. Some of the studies tested in the range of 0.1 to 10 Hz because of the limitations of the instrument settings. Results of G′ and G″ are dependent on the range of frequency, for instance Kazemirad et al. revealed that the outcome patterns of high and low frequency were consistent but with a greater magnitude in lower frequency range [40]. The strain (γ) used by the studies ranged from 0.01% to 2% but only one study applied up to 1000% to determine the limit of the linear viscoelastic regime [42]. The phonation threshold pressure (PTP) quantifies the minimum lung pressure needed to generate the desired voice. It is one of the aerodynamic measurements for the vocal fold. This measurement was used by a single study [36] in this review. The results are summarized in Table 2.

### 3.4. Other Characterization Parameters

Other than the mechanical properties of the biomaterials, different features were tested to further support the feasibility of these biomaterials for vocal fold injection. A total of seven different tests were carried out. The most frequent tests include particle and pore size quantification [37,38,41,43,50]. Injection force was only measured by two studies in this review [41,50]. Swelling ratio [37,38] and drug release tests [39,50] were performed in two studies, while morphology and gelation time were only carried out by single study [37]. The results are summarized in Table 3.

### 3.5. Cellular Response

To further identify suitable biomaterials, in vitro studies must include evaluations to elucidate the cellular response when integrated with the biomaterials. In vitro outcomes measured in this review included cell adhesion [43], cell viability/compatibility [37,39,50], cell phenotyping [49] and production of cytokines [49]. Among the types of cell used were human vocal fold fibroblasts (HVFF), NIH/3T3 cells, macrophages, vocal fold fibroblasts (VFF), Hacat cells and mouse NIH-3T3 embryonic fibroblasts. Ravanbakhsh and co-researchers suggested a cell viability threshold of 70% [37]. However, Fu and co-researchers compared cell viability between the sets and control to quantify the cytotoxicity level of the biomaterials [39]. An interesting in vitro study by Coburn and co-researchers quantified the response of macrophages when cultivated with hydrogel and vocal fold fibroblasts [49]. The results are summarized in Table 4.

## 4. Discussion

Even with the recent advances in regenerative therapy, no treatment is able to reconstitute a paralyzed vocal fold. Therefore, researchers have widely studied alternative biomaterials to reconstruct the physical properties of the vocal fold [51]. Since the viscoelasticity of the vocal fold is closely related to the efficiency of phonation, it is a primary concern when developing injectable biomaterials for vocal fold augmentation. Furthermore, studies showed that different stiffnesses of hydrogel would have different impacts on microenvironmental responses such as cell adhesion and proliferation [52,53]. Therefore, the rheology study of injectable biomaterial is vital to understand its deformation (elasticity) and flow of matter (viscosity) [54].

Up to date, no reference study is able to quantify the viscoelasticity of human vocal folds precisely. This is because the viscoelasticity of the vocal fold is influenced by many factors such as age, gender, hydration level, disease status such as laryngeal nerve paralysis, mass lesions and fibrosis [55,56]. There are two ways to quantify the viscoelasticity of the vocal fold: linear and non-linear measurement. Linear measurement has been used widely and it is characterized by shear rheology which mainly is divided into two types: (1) rotational rheometry and (2) linear skin rheometry (LSR) [57]. Studies suggested that rotational rheometry generates more consistent results than LSR with 1% strain. Most of the studies in this review used linear methods to quantify viscoelasticity. Across the studies, various frequencies and strain rates were used, hence direct comparisons between the results were hard to perform. Torsional wave experiment (TWE) was suggested to quantify the linear behavior of viscoelasticity as it can overcome sample inertia at higher frequencies [58]. It is recommended to include phonation frequency by humans as conducted in the study by Kazemirad et al. [59]. The tested frequencies were in the range of 110 Hz to 260 Hz and 220 to 440 Hz for males and females, respectively. The current literature review did not provide a consistent range for the viscoelasticity value of the native vocal fold. For instance, Goodyer et al. suggested a value for males of 246 Pa to 3536 Pa and for females of 286 Pa to 3332 Pa by using the simple shear model method [55]. However, another study has refuted this [15], suggesting the viscoelasticity of the human vocal fold superficial layer was 5.0 kPa (ranging from 4.7 to 5.4 kPa) for the superior medial and 7.0 kPa (ranging from 6.7 to 7.3 kPa) for the inferior medial surface. Recently, the inaccuracy of linear measurement was raised, and non-linear measurement was proposed [60]. This study demonstrated the non-linearity properties of the human vocal fold cover and ligament at a high strain of 0.5 (50%) with frequencies of 175 Hz and 125 Hz [60]. Tissues in this biological system, which consists of different compositions with distinct densities and topologies, appear to be the main obstacle in developing a suitable biomaterial for the vocal fold [61]. Therefore, this review prompts the need to provide significant values of vocal fold viscoelasticity across reported studies by statistical justification. Moreover, most of the studies tested the mechanical properties of biomaterials using linear measurement, which may not reflect their similarities with the native vocal fold. Inconsistency of testing parameters across studies highlights the insufficiency of the benchmark and established guidelines in developing a suitable biomaterial with desirable viscoelastic properties.

Even though injectable biomaterials for vocal fold augmentation possess desired viscoelasticity, inflammation is a main concern as foreign body reaction could lead to serious complications such as airway edema [62]. Moreover, rare hypersensitivity cases occurred after injection of bovine collagen which can lead to more severe onset symptoms and require medical attention for a longer period [63]. Not to mention that a previously well-known injectable biomaterial, Teflon (Du Pont, Wilmington, DE, USA), resulted in granuloma formation [64]. Granuloma formation is closely associated with chronic inflammatory response [65]. HA is widely used nowadays, but an unusual allergy response has been observed [66]. Therefore, preliminary data on cell immune response is much needed when evaluating a biomaterial in an in vitro study. The vocal fold consists of fibroblasts which have been proven to be closely associated with inflammatory response [67]. Inflammation in vocal fold is one of the pathological factors leading to vocal fold scarring [68]. As biomaterials are injected into the biological system, immune cells such as leukocytes detect them as foreign particles. The different chemistry and exterior characteristics of hosts can trigger an immune response [69]. During this immune response, pro-inflammatory cytokines such as TNF-α, IL-6, IL-8, IL-1β and MCP-1 will be liberated whereas anti-inflammatory cytokines such as IL-10 and IL-12 play crucial roles in quantifying the efficiency of eliminating harmful materials while providing protection to host [67,70]. During wound repair in the vocal fold, increased pro-inflammatory cytokines IL-1β and IL-6 by neutrophils and macrophages promote fibrosis. However, the anti-inflammatory cytokine, IL-10 could have the potential to dampen the inflammation [71]. Nevertheless, this review shows that only a single study has explained inflammatory responses when developing biomaterials [49]. This test is suggested to be included for future research to elucidate molecular mechanisms of inflammation when a novel biomaterial is developed for tissue regeneration. Both pro- and anti-inflammatory responses should be quantified to ensure the balanced inflammatory response after injection of biomaterial.

Apart from the above-mentioned tests, biomaterials with no migration and low resorption are keys to determine practicality for injection application in the clinic [72]. Injection of HA was observed to have low complication rates between 3 and 5%, mainly from inflammatory reactions [66,73]. This response might be due to foreign body reaction or contamination during the injection process. Biodegradation is explained when disintegration of biomaterial occurs in the biological system [74]. Clinical measurement of biodegradation can be achieved by resorption rate of the biomaterial [75]. Current commercial biomaterials are divided into two categories, short term and long term injectates [76]. Bovine-based gelatin and collagen products, human-based collagen, HA and carboxymethylcellulose have temporary effects whereas calcium hydroxyapatite (CaHA), Teflon (Du Pont, Wilmington, DE, USA), autologous fat and polydimethylsiloxane possess longer augmentation effects. Occasionally, clinicians opt for temporary augmentation aiming for natural recovery and reducing the need for medialization laryngoplasty [77]. Temporary augmentation is also recommended during the wait of reinnervation period [78,79]. However, clinical studies have suggested 15% to 20% over injection of short-term biomaterial to counter resorption, especially of water-based gels [80]. Biodegradation can be quantified by using hydrolysis by enzymes such as lysozyme and collagenase in certain natural materials such as chitosan, gelatin and alginate [81,82,83]. Nevertheless, none of the reviewed articles include biodegradation tests. Hence, this review suggests a degradation test as prolonged degradation rates of biomaterials could positively influence local cellular activities [84]. Further investigation of the composition after degradation should be carried out to study the cytotoxicity effect [85]. Accordingly, the end-products after degradation should be elucidated to provide evidence of background study during the initial stage of biomaterials development, specifically during in vitro studies. This could assist in providing strong evidence for future investigation such as in vivo and clinical studies.

Other characterization could be carried out depending on the specific requirements of hydrogel. The time taken for the hydrogel to polymerize should be optimized and 20 min was recommended by Ravanbaksh et al. [37]. Short gelation time could prevent destabilized gel washout from the desired location. On the other hand, it could result in insufficient injection time for clinicians [85,86]. A previous study reported that swelling ratio influenced the regulation of chondrocyte [87]. Thomas et al. conducted a swelling ratio test to determine the stability of the biomaterial to remain its original state when immersed in excess fluid as a simulation of the biological environment [53]. The porosity of biomaterials influenced the effectiveness of substances’ migration and mechanical properties. Therefore, fine-tuning between porosity and mechanical properties is needed to obtain effective biomaterials [88]. Homogeneous distribution of pore size has been shown to reduce cell interaction with the scaffold [89].

Among the biomaterials included in this review, some had been studied in clinical stages including micronized alloderm (Cymetra) (Lifecell Corp, Branchburg, NJ, USA) HA, CMC, collagen, Rofilan (Laborata es Filorga, Lisbonne, Paris, France), Radiesse (Merz, Franksville, WI, USA) and Restylane (Galderma Laboratories, Fort Worth, TX, USA) [27,29,66,90,91,92,93,94]. It is vita l to refer to the outcome of the clinical studies, so that future study can address the suggested drawbacks of these biomaterials. The drawbacks included: (1) inconvenient preparation steps and the need for over-injection for Cymetra (Lifecell Corp, Branchburg, NJ, USA) [90]; (2) inflammatory reaction and a lack of long-lasting effect in application of HA [29,66,94]. CMC and collagen had similar issues, making them suitable only for short term augmentation [91,92] and lastly (3) Rofilan (Laborata es Filorga, Lisbonne, Paris, France) was reported to show no improvement in voice acoustic analysis such as noise to harmonic ratio [93]. With that, this review suggests future study ought to include parameters that can address the limitations of these current biomaterials mainly with respect to longevity, inflammatory properties and viscoelasticity of biomaterials.

The findings from this review are limited by potential bias. In this study, only in vitro study was chosen as an inclusion criterion, leading to narrower results. Justification of this criterion is that this study aims to illuminate the importance of the initial stage of biomaterial development. Secondly, due to lack of facility to translate foreign languages, non-English literatures were excluded from this study. In addition, grey literature articles were also not evaluated in this work. We have, however, attempted to exclude duplicate articles in this review. To enhance the outcome of this review, statistical analysis is suggested for future evaluation. However, in vitro study should be a strong molecular support when exploring a higher level of studies.

## 5. Conclusions

In conclusion, there is a lack of benchmarks to standardize the evaluation of novel biomaterials for vocal fold injection. After summarizing the studies included in this review and comparing the study outcome measures used with available clinical outcomes, it is suggested to prioritize characterization of the viscoelasticity (to improve voice outcomes), inflammatory response (to reduce side effects) and biodegradation (to improve longevity) profiles of biomaterials. Even though results generated by in vitro studies may not be consistent with outcomes seen in in vivo and clinical studies, it should provide a fundamental insight to consider the suitability of biomaterials for further study. If the outcomes of both in vitro and in vivo studies support each other, it could strengthen conviction and provide strong evidence for pre-clinical studies.

## Figures and Tables

**Table 1:** Search strategy for all databases. (*: to obtain both singular and plural forms of the search criterion).

No.	Terms
1	Biomaterial*
2	Material*
3	Regenerative therap*
4	Hyaluronic acid*
5	Vocal fold injection*
6	Glottic insufficienc*
7	Vocal fold medialization*
8	Vocal fold augmentation*
9	Or/1–4
10	Or/5–8
11	And/9 & 10
12	Line 11: Restrict to period: January 2010 to March 2021

**Table 2:** Rheological study.

Author, Year	Type of Biomaterials	Study Measure Outcome	Summary of Results	Conclusion
1. Klemuk et al., 2010 [36]	1. Carbomer hydrogel 2. Micronized dermal graft tissue 3. Crosslinked HA hydrogel 4. HA and gelatin crosslinked hydrogel	(a) Rheology measurement by rotational (for 0.1 to 100 Hz) and piezoelectric rheometer (up to 2000 Hz): shear elastic (G′) and viscous moduli (G″) (linear) (b) Pressure threshold projection (PTP) vocal fold length and frequency: (i) For males: 15.8 mm and 125 Hz (ii) For females: 10.63 mm, 200 Hz	Shear elastic (G′) Mean values: 100 to 10,000 Pa Viscous moduli (G″) Mean values: 10 to 5000 Pa Loss tangent (G″/G′) in 100 to 1000 Hz Hylan B, Extracel and Cbmr: 0.08 to 0.66 (widest range) PTP value relative to nominal PTP value (0.283 kPa) Sample 2 had highest value (3× to 21×) and sample 4 had lowest value (0.01× to 0.05×)	Crosslinked HA hydrogel, HA and gelatin crosslinked hydrogel and carbomer hydrogel are suitable for voice production.
2. Mahboubi, Mohraz & Verma 2016 [46]	1. CaHA 2. CMC	(a) Viscosity modulus (ƞ) by rotational rheometer (b) Shear elastic (G′) and loss moduli (G″) with frequency sweeps at 0.01% strain and 0.1 to 200 Hz by oscillatory rheometer (linear)	Viscosity CaHA (43,100 Pa.s) was 10 times more viscous than CMC (4540 Pa.s). Heating and shearing G″ for CaHA reduced by 52%	Heating and shearing potentially reduces viscosity of CaHA.
3. Kimura, Mau & W Chan 2010 [47]	1. 3% bovine collagen (atelocollagen) 2. micronized Alloderm 3. CaHA 4. 2.4% crosslinked HA gel	(a) Elastic shear modulus (G′) tested with 0.3 to 0.5 mm gap size, 1 to 2% strain and 1 to 250 Hz. (b) Dynamic viscosity (n′) by custom build, controlled strain, linear simple shear rheometer system	Elastic shear modulus (G′) Atelocollagen performed the nearest value (about 1000 Pa) to the vocal fold superficial layer′s value. Dynamic viscosity (ƞ′) Atelocollagen performed the nearest value (about 0.7 Pa.s) to the vocal fold superficial layer′s value at around 135 Hz.	All biomaterials had stiffer properties compared to earlier studies, hence suggested for deep injection into the vocal fold but not into lamina propria.
4. Ravanbakhsh et al., 2019 [37]	1. Carboxylic (COOH) multi-walled functionalized carbon nanotube (CNTs) 2. Hydroxylic (OH) multi-walled functionalized CNTs	Storage modulus tested at 0.1 to 10 Hz and 1000 µm gap size, <5% shear strain by rotational rheometer (linear)	Storage modulus* - Increased with higher CNT concentration - OH-CNT had higher storage modulus than COOH-CNT	Mechanical strength of hydrogel was not influenced by the concentration of CNT.
5. Kim et al., 2015 [38]	1. Commercial HA 2. Unequal particle-sized middle viscosity HA (3,000,000 cP) 3. Unequal particle-sized low viscosity HA (30,000 cP)	Elasticity at 0.02 Hz (not specified)	Elasticity Commercial HA: 200 to 400 Pa Mid HA: 300 Pa Low HA: 3 Pa	Unequal particle-size HA showed better outcomes than commercial HA *in vivo*.
6. Choi et al., 2012 [48]	1. Rofilan (non animal stabilized biomaterial) 2. Restylane (double crosslinked 3. Dextran beads in HA (dextran microspheres)	(a) Shear viscosity (ƞ) (b) Mean elastic modulus (G′) (c) Mean viscous modulus (G″) at frequency of 0.1 to 10 Hz with strain controlled rheometer (linear)	Steady state viscosity (ƞ) Restylane had the highest (19.138 Pa∙s) value. Mean elastic modulus (G′) Reviderm had the highest (464.1 Pa∙s) value. Mean viscous modulus (G″) Reviderm had the highest (167.8 Pa·s) value.	All HA-based hydrogels had similar shear viscosities and the values were higher than reported human vocal fold but in vivo study showed that HA-based hydrogels were compatible with viscoelasticity of rabbit vocal fold.
7. Chan et al., 2014 [50]	1. PEG microparticles (MP) 2. Gelatin methacrylate MP 3. HA-methacrylate (HAMA) MP 4. Semi-IPN MP of HAMA & gelatine	Shear storage modulus (G′) and shear loss modulus (G″) at 0.6% strain by rotational rheometer (linear).	Viscoelasticity for PEG-DA:PEG When the ratio of PEG-DA:PEG increased 50 to 100%, G′ increased from 523 Pa to 1599 a; G″ increased from 38 Pa to 111 Pa	Photopolymerization method was able to synthesize soft MP with varying stiffness which was independent of its size.
8. Coburn et al., 2020 [49]	Glycol-chitosan hydrogel with different crosslinker concentrations: 0.005%, 0.01% and 0.02%	Mechanical characterization at 0.1 to 100 rad/s by rotational rheometer (linear)	Storage modulus (G′) and loss modulus (G″) 0.005%: around 50 and 18 Pa 0.01%: around 340 and 17 Pa 0.02%: around 740 and 15 Pa G′ value > G″: showing elastic property	Hydrogel with higher stiffness potentially caused inflammation but delayed expression of IL-10 at 72 h caused higher macrophage apoptosis.
9. Fu et al., 2015 [39]	Pluronic F127 with collagen of 1%, 2% and 3%	(a) Storage modulus (b) Loss modulus Rheology measurement at 1.0 rad/s and 0.5% strain by rotational rheometer (linear)	Elastic modulus (G′) at 1.0 rad/s Pluronic F127 with highest collagen (3%) exhibited lowest G′ (94.0 kPa). Viscous modulus (G″) at 10 to 100 rad/s* Pluronic F127 with collagen did not reduce sharply compared to without collagen.	Pluronic F127 with collagen enhanced the drug release time and favoured cell growth.
10. Kazemirad, Heris & Mongeau 2016 [40]	1. Sample 1 to 3: 0.50%HA + crosslinker PEGDA of 0.25%, 0.5% & 1.0% 2. Sample 4 to 5: 0.45%HA + 0.05% gelatin (Ge) of 0.1% and 0.2%	(a) Shear storage (G′) (b) Loss moduli (G″) at frequency up to 4000 Hz (linear)	Shear storage (G′) & Loss moduli (G″) Sample 4 (G′: 19.61 Pa; G″: 5.00 Pa) and 5 (G′: 12.24 Pa; G″: 8.50 Pa) were comparable to viscoelasticity with human vocal fold.	With optimized concentration of HA, Ge and crosslinker, the hydrogel showed comparable viscoelasticity.
11. Brown et al., 2019 [41]	Silk protein-based in HA suspension	Mechanical properties at 0.1 to 10.0 Hz and 1% strain by dynamic rotation shear rheometer (linear)	Mechanical properties - Silk suspension increased stiffness less rapid (5× lesser) than CaHA-CMC - Injection of silk suspension (1.5 times) yield lesser stiffness in vocal fold than CaHA-CMC (4.0 times)	Silk-HA had similar viscoelasticity properties with porcine vocal fold.
12. Li et al., 2018 [42]	Chemically crosslinked resilin-like-polypeptide (RLP) hydrogel: 1. Sample 1: 10 wt% 2. Sample 2: 15 wt%	(a) Shear storage modulus at 0.1 to 100 rad/s (b) Storage moduli (G′) and loss moduli (G″) by stress controlled rheometer (c) strain sweep test of 0.01% to 1000% (linear)	Shear storage modulus Sample 1 and 2 increased rapidly until 1000 Pa and 2000 Pa respectively Storage moduli (G′) and loss moduli (G″) G′ was higher than G″ for 100 to 200 fold. Strain sweep Sample 1: 265% Sample 2: 245% High resistance to break.	Rheological properties of the hydrogels were in the range of native vocal fold tissue.
13. Pruett et al., 2020 [44]	Fabrication of microporous annealed particle (MAP) by water-in-oil emulsion	Young’s modulus (linear), compare with vocalis muscle	Young’s modulus 1.9 wt% MAP showed with porcine vocal fold’s muscle comparable (~15,000 Pa).	MAP gel exhibited similar rheological properties with porcine vocal fold tissue.
14. Karajanagi et al., 2011 [45]	PEG30 hydrogel	Elastic shear properties (G′) from 1 to 10 Hz with 0.6% strain by rotational rheometer (linear)	Viscoelasticity* PEG30 showed softer hydrogel compared to value reported in literature review.	PEG30 demonstrated optimal physical properties for vocal fold injection.

Remarks: Result with * indicated no reported value.

**Table 3:** Other characterization parameters.

Author, Year	Type of Biomaterials	Study Measure Outcome	Summary of Results	Conclusion
1. Ravanbakhsh et al., 2019 [37]	1. Carboxylic (COOH) multi-walled functionalized CNTs 2. Hydroxylic (OH) multi-walled functionalized CNTs	(a) Morphology (b) Pore size (c) Swelling ratio (d) Gelation time	Pore size Diameter of CNTs: 45 ± 5 nm Increased by 33% with increased concentration of COOT-CNT but not significant in OH-CNT. Swelling ratio Increased by 5% with increased COOT-CNT concentration. OH-CNT had no effect on this property. Gelation time* Increased starting 750 μg/mL of CNT	COOH-CNT hydrogel showed larger pore size which might enhance cell migration.
2. Kim et al., 2015 [38]	1. Commercial HA (Restylane) 2. Unequal particle-sized middle viscosity HA 3. Unequal particle-sized low viscosity HA	(a) Particle size (b) Swelling ratio	Particle size Restylane: 200 µm Mid HA: 300 to 500 µm Low HA: No size Swelling ratio Restylane: 100 to 200% Mid HA: 130% Low HA: 200%	Unequal particle-size HA showed better outcomes than Restylane^®^ in vivo.
3. Chan et al., 2014 [50]	1. PEG MP 2. Gelatin methacrylate MP 3. HA-methacrylate (HAMA) MP 4. Semi-IPN MP of HAMA & gelatin	(a) Particle size (b) Drug release test	Ability to inject Can be injected through 22 gauge needle Particle size (D90) Range from 136 µm to 162 µm MP produced had uniform particle distribution with ~1.5 polydispersity (PDI). Increased stirring speed up to 600 rpm or surfactant reduced the size from 515 to 140 µm. Drug release test* Drug encapsulation and release in PEG NS/MP was lower than from NS alone.	Higher stirring speed and surfactant concentration reduced size of MP and drug release time.
4. Fu et al., 2015 [39]	1. Pluronic F127 with collagen of 1%, 2% and 3%	(a) Morphology (b) Drug release test	Pore size With increased concentration of collagen incorporated in Pluronic F127, pore size was increased (from 5–20 µm to 20–40 µm). Drug release Collagen incorporated in Pluronic F127 reduced drug (ofloxacin) release (43.6% to 48.1%).	Pluronic F127 with collagen enhanced the drug release time and favoured cell growth.
5. Brown et al., 2019 [41]	Silk protein-based in HA suspension	(a) Pore size (b) Injection force through 24 G long needle and 50 cm catheter with 1.05 mm inner diameter at 13 mm/min speed	Pore size Diameter ranging 10 to 100 um and had ability to return into original shape after compression. Injection force Silk protein (34.9 N) needed less force than CaHA-CMC (51.4 N) as control.	Particle size of silk-HA allowed macrophage passage, tissue adherence and was biocompatible.
6. Chung et al., 2017 [43]	PDMScoated with PDA	(a) Particle size (b) Morphology	Particle size 79.23 µm ± 2.23 with 2.81% coefficient of variation. (less than 5% showed highly uniform size distribution) Morphology PDMS microsphere with PDA had a rougher surface while without PDA had a smoother surface.	PDMS was injectable, non-absorbable and showed better cell adherence.

Remarks: result with * indicates no reported value.

**Table 4:** In vitro study.

Author, Year	Type of Biomaterials	Study Measure Outcome	Summary of Results	Conclusion
1. Ravanbakhsh et al., 2019 [37]	1. Carboxylic (COOH) multi-walled functionalized CNTs 2. Hydroxylic (OH) multi-walled functionalized CNTs	Cell viability of HVFF	Cell viability 1. COOH-CNT up to 750 ug/mL; 2. OH-CNT up to 1250 ug/mL as the cytotoxicity level increased after this threshold.	COOH-CNT had higher cytotoxicity than OH-CNT
2. Chan et al., 2014 [50]	1. PEG MP 2. Gelatin methacrylate MP 3. HA-methacrylate (HAMA) MP 4. Semi-IPN MP of HAMA & gelatin	Cytocompatibility test on NIH/3T3 cells	Cytocompatibility test 0.1 to 50 mg/mL PEG50 culture resulted in cell viability of 80%.	Hydrogel produced by photopolymerization was cytocompatible.
3. Coburn et al., 2020 [49]	Glycol-chitosan hydrogel with concentration: 1. 0.005% 2. 0.01% 3. 0.02%	(a) Production of cytokines by macrophage (b) Macrophage viability (c) Macrophage phenotyping: -CD11b (+) CD33/CD80:proinflammatory CD33/CD206:anti- inflammatory	Production of cytokines TNF-α and IL-10 increased in hydrogel culture and with increased stiffness of hydrogel. Cell viability Macrophage viability was reduced in hydrogel culture. Cell phenotyping More CD33/CD206 (anti-inflammatory) expressing macrophage in macrophage+ VFF+hydrogel than macrophage+hydrogel.	Hydrogel with higher stiffness potentially caused inflammation but delayed expression of IL-10 at 72 h caused higher macrophage apoptosis.
4. Fu et al., 2015 [39]	Pluronic F127 with collagen of 1%, 2% and 3%	Cell viability (Hacat cells)	Cell viability Collagen incorporated in Pluronic F127 improved cell adhesion and viability.	Pluronic F127 loaded with collagen improved cell adhesion and viability.
5. Chung et al., 2017 [43]	PDMS PDA	Cell adhesion test (mouse NIH-3T3 embryonic fibroblast)	Cell adhesion test PDMS microsphere with PDA had more cells attached on its surface.	PDMS with PDA demonstrated better cell adherence.

## Data Availability

Data sharing is not applicable to this article as no new data were created or analyzed in this study.

## Abbreviations

CaHA	Calcium Hydroxypatite
CMC	Carboxymethylcellulose
CNTs	Carbon nanotube
COOH	Carboxylic
COX2	Cyclooxygenase
ECM	Extracellular matrix
HA	Hyaluronic acid
HAMA	HA-methacrylate MP
HVFF	Human vocal fold fibroblast
IL-1β	Interleukin-1 beta
IFN-γ	Interferon-gamma
LSR	Linear skin rheometry
MAP	Microporous annealed particle
MAPK	Mitogen-activated protein kinase
MCP-1	Monocyte chemoattractant protein-1
MP	Microparticles
NF-ĸB	Necrosis factor kappa beta
OH	Hydroxylic
PDA	Polydopamine
PDMS	Poly(dimethysiloxane) microspheres
PEG	Polyethylene glycol-diamine
PEGDA	Poly(ethylene glycol) diacrylate
PTP	Phonation threshold pressure
RLN	Recurrent laryngeal nerve
RLP	Resilin-like-polypeptide
TGF-β	Transforming growth factor-beta
TNF-α	Tumor necrosis factor-alpha
TWE	Torsional wave experiment
